# Atteinte hépatique au cours de la maladie de Rendu-Osler: à propos d’un cas et revue de la littérature

**DOI:** 10.11604/pamj.2016.24.326.9816

**Published:** 2016-08-25

**Authors:** Hanen Loukil, Mouna Snoussi, Hela Fourati, Faten Frikha, Raida Ben Salah, Moez Jallouli, Sameh Marzouk, Zeineb Mnif, Zouhir Bahloul

**Affiliations:** 1 Service de Médicine Interne CHU Hédi Chaker Sfax, Tunisie; 2 Service de radiologie CHU Hédi Chaker Sfax, Tunisie

**Keywords:** Rendu Osler, liver involvement, cholestasis, Rendu Osler, liver involvement, cholestasis

## Abstract

Patiente âgée de 48 ans était hospitalisée pour une cholestase asymptomatique hépatique. Elle rapportait une histoire personnelle et familiale d’épistaxis récidivante. Le bilan biologique révélait une anémie ferriprive et une cholestase modérée. Les sérologies virales ainsi que les anticorps anti tissu hépatique étaient négatifs. Le scanner abdominal objectivait de multiples shunts artério-veineux dans la région sous-capsulaire du foie. Le diagnostic d’une atteinte hépatique dans le cadre d’un Rendu Osler était retenu. Un traitement martial était prescrit et une surveillance biologique et morphologique du foie était entreprise.

## Abstract (en)

A 48 years-old-woman was admitted for anectric cholestasis. A history of recurrent personal and familial epistaxis was noted. Biologic findings revealed iron deficiency anemia and moderate cholestasis. Viral serologic tests, antimitochondrie and anti smooth muscle antibodies were negative. Abdominal tomography showed multiple arterio-venous shunts of the liver. The diagnosis of liver involvement due to Rendu Osler disease was made. Treatment with oral ferrous iron of 150 mg/day was administered and regular biological and morphologic controls of liver was decided.

## Introduction

La maladie de Rendu-Osler (MRO) ou télangiectasie hémorragique héréditaire est une maladie autosomique dominante rare. C’est une angiodysplasie multisystémique caractérisée par la présence de télangiectasies cutanéo-muqueuses souvent hémorragiques et de malformations arterio-veineuses viscérales essentiellement hépatiques, pulmonaires et neurologiques centrales [1]. Nous rapportons l´observation d´une patiente ayant présenté une cholestase anictérique révélatrice d´une MRO.

## Patient et observation

**Figure 1:**
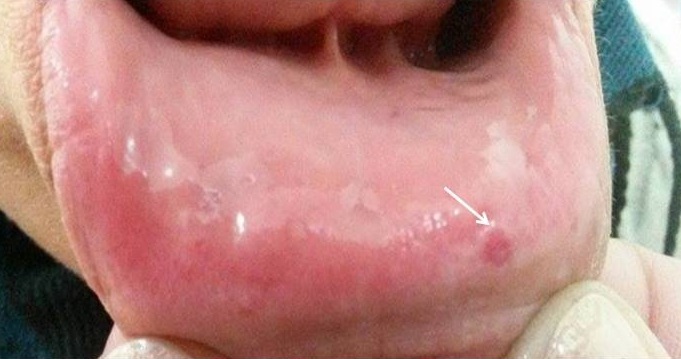
Télangiectasie de la lèvre chez la patiente

**Figure 2:**
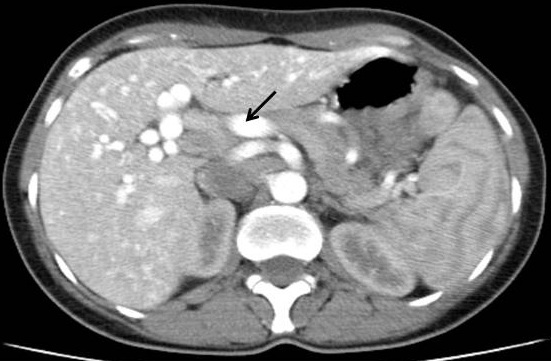
Aspect dilaté de l’artère hépatique (flèche), ainsi que des artères hépatiques droite et gauche

**Figure 3:**
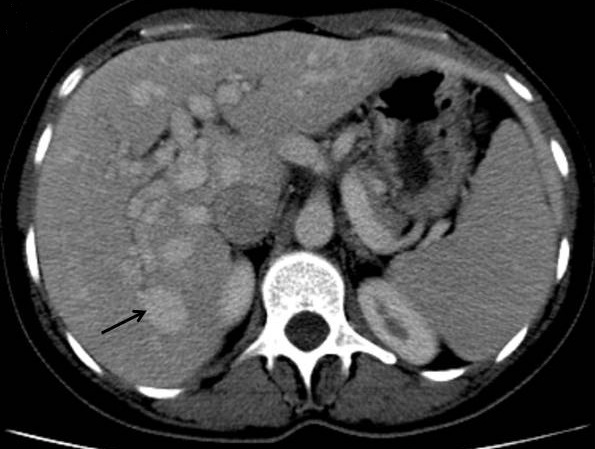
Malformation artério-veineuse intra hépatique (flèche) entre une branche de l’artère hépatique et une branche de division d’une veine hépatique

Madame S.H, âgée de 48 ans, était hospitalisée dans notre service au mois de juin 2014 pour exploration d’une anémie associée à une perturbation du bilan hépatique. Ses antécédents étaient marqués par des épisodes d’épistaxis de faible abondance itératifs apparus depuis l’âge de 6 ans. La même symptomatologie était rapportée chez le frère. A l´examen clinique, l’état général était conservé, il existait une sensibilité de l’hypochondre droit, une hépatomégalie avec une flèche hépatique à 15 cm et des lésions de télangiectasies sur la lèvre (Figure 1) et la langue. L’examen ORL spécialisé objectivait de multiples télangiectasies de la muqueuse nasale. A la biologie il n y´avait pas de syndrome inflammatoire (la vitesse de sédimentation était à 25 mm à la première heure et la protéine C réactive à 3 mg/L (normale < 5 mg/l. L´hémogramme montrait une anémie hypochrome microcytaire à 5 g/dl. La férritinémie était basse à 1,4 µg/l. Le bilan hépatique objectivait une cholestase hépatique avec des gammaglutamyltranspeptidases à 225U/L (5 fois la normale) et des phsphatases alcalines à 400U /l (4 fois la normale). L’alanine aminotransférase était à 35 U/L (VN <40U /l) et l’aspartate aminotransférase à 30U /L (VN<40U /l). L’électrophorèse des protéines plasmatiques et le taux de prothombine étaient normaux. Devant cette cholestase modérée, une enquête étiologique était entreprise : les sérologies virales de l’hépatite B et C étaient négatives. Le bilan immunologique comportant les anticorps antinucléaires, les anticorps anti-mitochondrie, anti-muscle lisse et anti-LKM1 et 2 était négatif. L’échographie abdominale objectivait une hépatomégalie homogène associée à une dilatation des veines sus hépatique. La tomodensitométrie thoraco-abdominale mettait en évidence une artère hépatique dilatée avec de multiples shunts artério-veineux dans la région sous-capsulaire du foie (Figure 2 et Figure 3).

**Figure 4:**
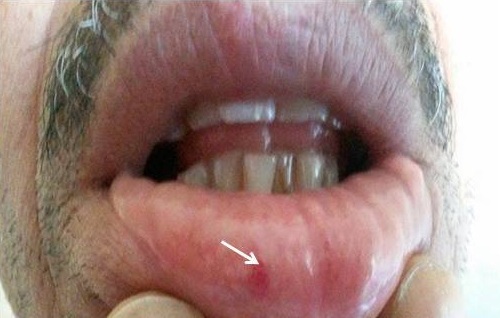
Télangiectasie de la lèvre chez le frère

Le diagnostic retenu était celui d’une MRO compliquée d’atteinte hépatique devant: l’épistaxis récidivante, les antécédents familiaux d’épistaxis et de télangiectasies labiales chez le frère (Figure 4) et les données des examens morphologiques hépatiques confirmant les shunts artério-veineux hépatiques. Le bilan lésionnel de la maladie englobant une tomodensitométrie thoracique, une échographie cardiaque ainsi qu’une fibroscopie oeso-gastroduodénale étaient normales. La patiente était mise sous supplémentation martiale, une surveillance régulière aussi bien biologique que morphologique de l’atteinte hépatique était décidée. A son suivi, la cholestase hépatique était stationnaire et aucune autre complication n’était notée.

## Discussion

La MRO est une maladie génétique à transmission autosomique dominante, à pénétrance variable. Elle est due à des mutations de gènes impliqués dans les voies de signalisation du TGF dans la cellule endothéliale à l’origine d’une hyperprolifération endothéliale [1]. Ce trouble de l’angiogenèse se traduit par des épistaxis récidivantes dès le jeune âge, des télangiectasies cutaneo-muqueuses dès la 3^ème^ décennie et des malformations artério-veineuses essentiellement pulmonaires et hépatiques [2].

Les atteintes hépatiques constituent une complication potentiellement grave au cours de la MRO. L’angiodysplasie hépatique est caractérisée par des malformations artério-veineuses pouvant intéresser tous les vaisseaux du foie aboutissant à des shunts artérioveineux, artérioportaux et portoveineux [3]. Les lésions hépatiques sont de fréquence variable selon les moyens diagnostiques, 33-72% dans les séries échographiques et 67–74% dans les séries scannographiques et 90% dans les séries autopsiques [3, 4]. Les signes cliniques de l’atteinte hépatique au cours de MRO sont variés chez les patients selon la gravité des shunts hépatiques. Habituellement asymptomatique comme chez notre patiente et elle est révélée par une cholestase d’intensité variable selon la sévérité des malformations artério-veineuses parfois associée à une cytolyse modérée [5, 6]. Dans les cas sévères et compliqués, il peut s’agir d’une ascite, des œdèmes des membres inférieurs, des douleurs de l’hypochondre droit, une dyspnée et plus rarement une hémorragie digestive avec une anémie par rupture de varices œsophagiennes. L’examen physique peut mettre en évidence un Thrill hépatique et une hépatomégalie ainsi qu’une splénomégalie [7]. Pour le diagnostic positif, l’exploration radiologique est indispensable. La tomodensitométrie est l’examen clé pour diagnostiquer les anomalies de l’artère hépatique au cours de MRO. Il objective la dilatation de l’artère hépatique associée à une dilatation des veines hépatiques et portales [8]. L’angioscanner hépatique montre également les shunts artério-veineux et arterio-portaux [8, 9]. Les complications rapportées sont les hyperplasies nodulaires focales du foie, les lithiases intra-hépatiques et la cirrhose caractérisée histologiquement par une fibrose péri vasculaire [3].

Le traitement de l’atteinte hépatique de la MRO est difficile, dépendant de son retentissement et de sa gravité. Les patients asymptomatiques, tel est le cas de notre patiente, ne nécessitent aucun traitement [3]. En revanche, chez les patients présentant une hépatopathie compliquée d’insuffisance cardiaque à débit élevé, le traitement est symptomatique basé sur les diurétiques et les bétabloquants [1]. La transplantation hépatique reste le seul traitement curatif des formes sévères à retentissement cardiaques ou hépatiques majeures [10].

## Conclusion

L’atteinte hépatique est fréquente et potentiellement graves au cours de la MRO. Sa recherche par les explorations radiologiques et au mieux par la tomodensitométrie doit être envisagée au diagnostic de la maladie et pendant le suivi afin de retarder ses complications sévères.
